# Intimate Partner Violence and Subsequent Depression in Women: A Systematic Review and Meta‐Analysis of Longitudinal Studies

**DOI:** 10.1002/brb3.70236

**Published:** 2025-01-19

**Authors:** Christopher B. Watson, Vicki Bitsika

**Affiliations:** ^1^ Brain Behaviour Research Group University of New England Armidale Australia

**Keywords:** depression | intimate partner violence | meta‐analysis | systematic review

## Abstract

**Introduction:**

Intimate partner violence (IPV) and depression are global health concerns with high prevalence rates and substantial negative impacts on individuals and the wider community. Women are particularly vulnerable to both IPV victimization and depressive disorders, and both are recognized worldwide as priorities for women's health. The aim of this systematic review and meta‐analysis was to determine whether recent longitudinal empirical evidence supports exposure to IPV as a contributing factor to the subsequent onset of depression in women.

**Methods:**

A search was performed in August 2024 of the *Medline*, *PsychInfo*, and *EBSCOHost* databases for longitudinal studies published after the year 2013, and 1193 studies were identified. Studies were included if they were written in English and measured IPV as an independent variable with depression as a dependent variable. Studies were excluded if depression was not measured separately from other variables or did not report primary quantitative data. Eleven studies with 118,544 female participants met the inclusion criteria for review.

**Results:**

Ten of the 11 reviewed studies reported a statistically significant positive association between exposure to IPV and depression in women. A random effects meta‐analysis was used to generate pooled odds ratios from nine estimates, which demonstrated that female IPV survivors have significantly increased odds of developing subsequent depression (*OR* = 1.92, (95% CI: 1.28, 2.86); although, there was high heterogeneity across studies (*I* ^2^ = 98.3%, *p* < 0.001). Ten of the 11 studies were from high‐income, industrialized countries, which limits the global application of these findings.

**Conclusions:**

These findings suggest that IPV may be one of many contributing factors for depression in women. However, variability in the definition of IPV and inconsistent adjustment for confounders across studies limits firm conclusions. The findings of this review suggest that strategies to prevent IPV could play a role in reducing the prevalence of depression. They also support the inclusion of depression screening for survivors of IPV in clinical approaches and a review of the effectiveness of IPV‐related depression intervention strategies.

## Graphical abstract

The aim of this systematic review and meta‐analysis was to determine whether recent longitudinal empirical evidence supports exposure to intimate partner violence as a contributing factor to the subsequent onset of depression in women. Findings suggest a positive association; however, high heterogeneity across studies limits firm conclusions.

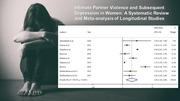

## 1 Introduction

Research suggests that intimate partner violence (IPV), which is defined as physical, sexual, or psychological abuse by an intimate partner (WHO 2021), may play an important role in women's depression as a traumatic life stressor (Ahmadabadi et al. 2020; Daugherty et al. 2021; White et al. 2024). The connection between stress and depression has been well supported by neurobiological studies of the stress responsivity pathways (Belleau, Treadway, and Pizzagalli 2019; Cunningham et al. 2021 Gold 2015; Park et al. 2019) and evidence suggests that IPV is a highly stressful and increasingly common phenomenon (Sardinha et al. 2022; Yim and Kofman 2019). Both biological and psychological studies of IPV report that it causes the stress‐related endocrine and immune‐inflammatory dysregulations and psychological stress responses that have been linked to depression (Alhalal and Falatah 2020; Chen et al. 2023; Yim and Kofman 2019). Women are twice as likely as men to experience IPV with an estimated global lifetime prevalence of 30% (WHO 2019a). Despite this, evidence on the relationship between experiencing IPV and the subsequent onset of depression in women remains unclear.

Depression is the largest contributor to years lived with a disability worldwide (Moreno‐Agostino et al. 2021). It is estimated that up to 5% of the world's population will experience depression in a 12‐month period and that up to 30% will experience an episode in their lifetime (World Health Organization [WHO] 2022). Previous research has demonstrated an increasing trend in prevalence (Liu et al. 2020; Ormel et al. 2020; Weinberger et al. 2018) despite the development of new treatments, improved access to mental health services, and a worldwide growth in the prescription of antidepressant medication (Alabaku et al. 2023; Ormel et al. 2022; WHO 2019b). This trend is particularly concerning for women's health, as females are twice as likely as males to experience depression in their lifetime and are also more likely to exhibit suicidal behavior associated with their depression (American Psychological Association [APA] 2022). Therefore, depression has been identified as a global mental health priority for women, and there is an urgent need to identify and address factors that may contribute to its rising prevalence (WHO 2022).

Several reviews of cross‐sectional studies have reported a bidirectional positive association between experiencing IPV and depression (Beydoun et al. 2012; Coker et al. 2002; Golding 1999; Lohmann et al. 2024). However, cross‐sectional analyses do not allow determination of a cause‐and‐effect relationship as they do not assess temporal priority or the association between variables over time. Comparatively, longitudinal studies can establish causation through the repeated measurement of variables and the examination of their interactions over prolonged periods. However, there are very few reviews examining the longitudinal relationship between IPV victimization and the subsequent onset of depression. The most recent of these reviews was performed by Devries et al. (2013) on findings published prior to 2013. That review reported on a random effects meta‐analysis of six effect estimates to calculate a pooled odds ratio which demonstrated that exposure to IPV almost doubles the odds of a new onset of depressive symptoms (OR = 1.97 [95% CI 1.56–2.48]).

Given the paucity of recent reviews, the cross‐sectional evidence of an association between IPV and depression, and the importance of IPV and depression to global women's health outcomes, an examination of post‐2013 longitudinal studies focused on the relationship between IPV exposure and depression was warranted. Therefore, the primary aim of this systematic review and meta‐analysis was to determine if post‐2013 empirical evidence supports earlier findings that IPV victimization has a positive relationship with the development of depression in women. Given the findings of Devries et al. (2013), and the well‐established correlation between stress and depression, it was hypothesized that IPV victimization would contribute to the subsequent development of depression in women.

## 2 Method

### 2.1 Search Procedure

To identify longitudinal studies of the association between IPV and depression, a search was performed in August 2024 in the *Medline*, *PsychInfo*, and *EBSCOHost* databases. Studies were considered longitudinal if depression was measured at more than one time point greater than 12 months apart. The search terms used were “intimate partner violence,” “domestic violence,” “partner abuse,” “depression,” “depressive disorder,” “depressive symptoms,” “major depressive disorder,” and “longitudinal.” In addition, advanced options were selected to allow each database to apply related words and equivalent subjects to the search. The search was limited to studies published post‐2013 to assess if recent evidence supports the findings of previous comparable meta‐analyses, such as the report by Devries et al. (2013). All identified studies were entered into a Microsoft Excel spreadsheet, and duplicates were removed.

**FIGURE 1:**
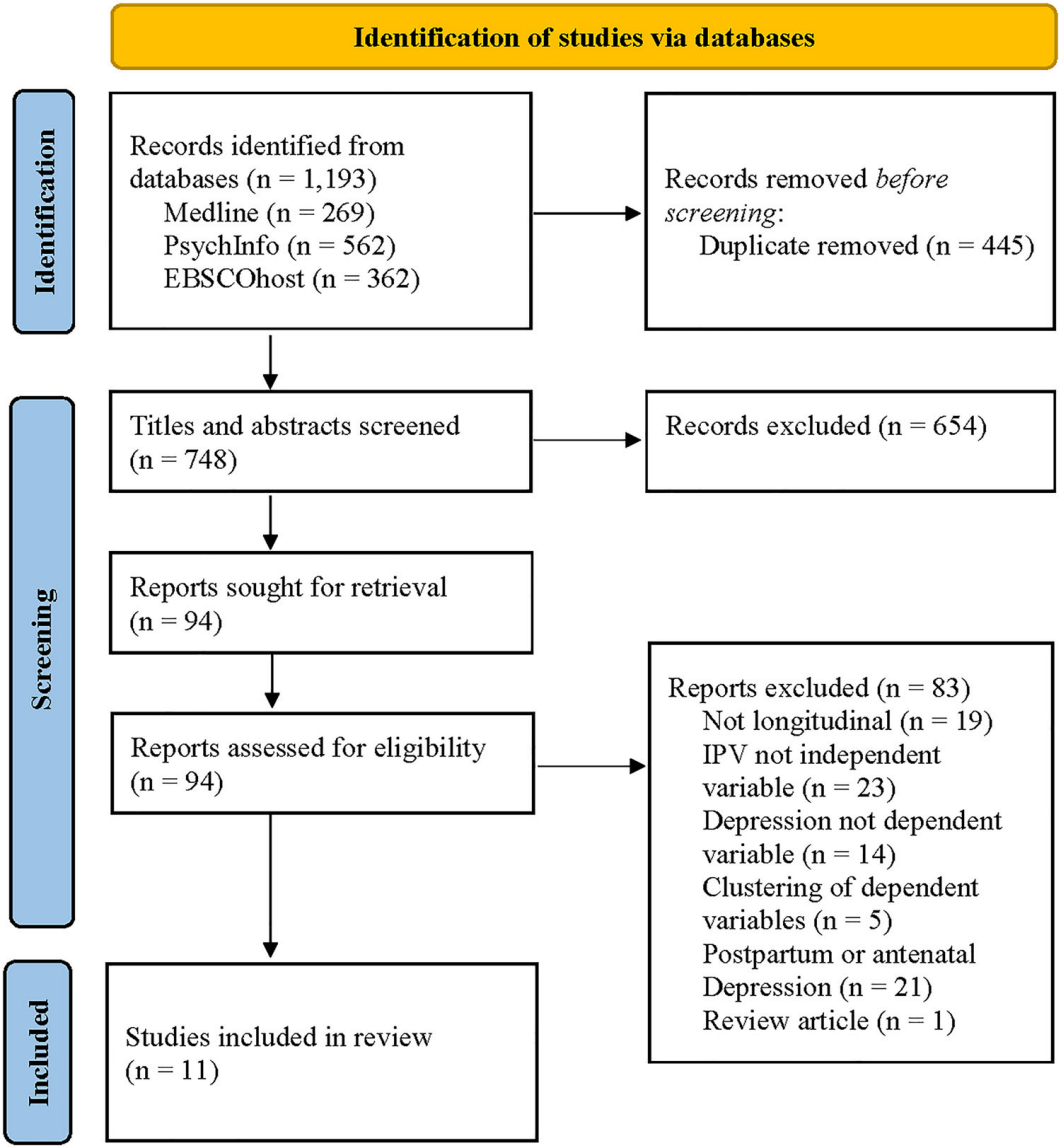
**|** PRISMA flowchart of search and selection process for studies examining the relationship between intimate partner violence and depression.

Before applying the inclusion and exclusion criteria, study titles and abstracts were reviewed by the first author to ensure alignment with the research topic. In addition to the database search, a manual search was performed by identifying possible relevant studies from the citations of those identified in the initial search. This manual search did not produce any additional studies relevant to the review. Figure 1 shows the number of articles identified from each of the databases and the selection process undertaken.

### 2.2 Exclusion, Non‐Exclusion and Inclusion Criteria

After the initial screening of study titles and abstracts, full texts were sourced for papers that aligned with the criteria set for the review. Studies were excluded if they were not written in English and not published in a peer‐reviewed journal. Reviews, case studies, opinion articles, books, qualitative studies, and research that did not report primary quantitative data for IPV and depression were also excluded. Studies in which depression was not measured separately from a comorbidity, such as post‐traumatic stress disorder or anxiety, or assessed as part of an overall mental health assessment were also omitted. Studies were included in the review if they measured IPV as an independent variable with depression as the dependent variable. Only effect estimates of female populations were considered in line with the scope of this systematic review and meta‐analysis.

### 2.3 Data Extraction and Quality Assessment

Descriptive information such as the number of female participants, the IPV and depression measures used, the frequency of measurement, the length of time between baseline measurement and follow‐up, and adjustments for confounders was extracted from the studies and entered in an Excel spreadsheet. Effect estimates assessing IPV as the independent variable and depression as the dependent variable in female samples were extracted for meta‐analysis. For this review, any data pertaining to male participants were removed, and this process occurred for eight studies.

Each study and its corresponding effect estimates were assessed for quality as determined by the use of IPV and depression measures with adequate reliability and validity, examination of the reference group formation for each study, and an assessment of the risk of bias for each study. Particular focus was placed on analyzing how the studies controlled for potential confounders given the variety of factors that were reported to contribute to depression and the episodic nature of IPV. Baseline measurements of depression and adjustment for socio‐demographic factors were key quality criteria for the calculation of the meta‐analysis.

### 2.4 Statistical Analysis

There was variation in the data collection and statistical analysis methods used across the reviewed studies. IPV and depression were measured using a number of approaches, including self‐report questionnaires, surveys, and diagnostic interviews. The studies also reported differing effect estimates, including odds ratios, incident relative risk, risk ratios, and correlation coefficients. For the purposes of the current meta‐analysis, odds ratios (adjusted and unadjusted) were extracted from the publications to calculate a pooled odds ratio using a random‐effects model. A pooled odds ratio was selected as the measure of effect size for two reasons: (1) the majority of reviewed studies presented their findings in odds ratios; and (2) to align with previous systematic reviews and meta‐analyses to allow for ease of comparison (Devries et al. 2013). Regression coefficients were converted to odds ratios using *R* software version 4.3.1, and confidence intervals were calculated. Meta‐analysis calculations were performed using the statistical software *Stata* version 15.0.

Several studies reported effect estimates for subtypes of IPV, such as physical violence, sexual violence and emotional violence. Overlap in the definition of these subtypes could result in the same measures being included multiple times in a single analysis (e.g., an act of sexual violence may also be considered physical violence). Subsequently, five conditions were set to avoid the double count of participants (1) if an overall IPV effect estimate was reported in addition to effect estimates for IPV subtypes, then only the overall estimate was used in the meta‐analysis, (2) if both unadjusted and adjusted effect estimates were reported, then the estimate that adjusted for the most confounders was included, (3) if no overall estimate was provided but an effect estimate for each sub type of IPV was reported, then these estimates were not included, (4) if a study provided multiple estimates on subsamples of participants, then all estimates were included (5) if a study reported an effect estimate for the same participants at two different timepoints, only the estimate with the smallest confidence interval was included.

In addition to the above, a meta‐analysis that included all effect estimates (subtypes and overall IPV) was performed. This process was used as a sensitivity analysis to examine if results were congruent with the outcome of the main analysis. All effect estimates relating to depression findings are presented in the description of reviewed studies below in Table 1.

**TABLE 1:** **|** Detailed description of studies examining the relationship between IPV and subsequent depression in women.

		Sample						
Author/s	Year	Location (Country)	Total females (N)	Source	Test of IPV	Test of depression	Type of IPV	Depression finding/s
Ahmadabadi et al.	2020	Australia	891	Birth cohort from the *Mater‐University of Queensland Study of Pregnancy*	CAS	CIDI	Any Physical Emotional Harassment	IPV survivors with no history of depressive disorder diagnosis at 21 years had increased odds of new onset depression at 30 years: Any maltreatment: ***aOR* = 2.70 (95% CI: 1.00, 7.40)** ^*^ Physical abuse only: *aOR* = 1.80 (95% CI: 1.10, 3.00) ^*^ Emotional abuse only: *aOR* = 1.50 (95% CI: 1.00, 2.60) ^*^ Harassment only: *aOR* = 1.30 (95% CI: 0.80, 2.30)
Cations et al.	2021	Australia	12,259	Participants from the *Australian Longitudinal Study of Women's Health*	Self‐reported history of IPV	GDS	Any	IPV survivors with no depression at baseline had increased odds for incident depression: ***aOR* = 1.36, (95% CI: 1.11, 1.67)** ^**^
Chandan et al.	2020	UK	92,735	Primary care records derived from *The Health Improvement Network* database	Primary care records	Primary care records	Any	IPV survivors with no depression at baseline had an increased incident risk rate for developing a depressive disorder: ***IRR* = 3.40 (95% CI: 3.16, 3.67)** ^***^
Chatterji and Heise	2021	Rwanda	1536	Data from the *Indashyikirwa* trial, a community‐randomized controlled trial	WHOMCS	CESD‐10	Physical Sexual Psychological	IPV survivors with no depression at baseline had increased risk ratios for depression 24 months later: Physical IPV: *aRR* = 1.44 (95% CI: 1.05, 1.98) ^*^ Sexual IPV: *aRR* = 1.45 (95% CI: 1.08, 1.96) ^*^ Emotional IPV: *aRR* = 1.82 (95% CI: 1.15, 2.89) ^*^ Physical and/or sexual IPV: *aRR* = 1.49 (95% CI: 1.13, 1.96) ** Severe physical and/or sexual IPV: *aRR* = 1.29 (95% CI: 1.01, 1.65) ^*^
Han et al.	2019	Korea	3143	Nationally representative sample from the *Korea Welfare Panel Study*	CTS	CESD‐11	Any Physical Verbal	IPV survivors with no depression at baseline had increased odds ratios for depressive symptoms 1 year later Any IPV—***aOR* = 1.18 (95% CI: 0.82, 1.70)** Physical IPV—*aOR* = 1.96 (95% CI: 1.31, 2.95) ^**^ Verbal IPV—*aOR* = 1.14 (95% CI: 0.78, 1.66)
Herbert et al.	2022	UK	1764	Data from the *Avon Longitudinal Study of Parents and Children*	PROVIDE	MFQ	Any	Exposure to IPV at baselines doubled the odds of developing depressive symptoms 2 years later: ***OR* = 2.10 (95% CI: 1.57, 2.81)** ^*^
Johnson et al.	2014	USA	662	Data from the *Toledo Adolescent Relationships Study*	CTS	CESD‐10	Any	Exposure to IPV had a positive association with depressive symptoms in females over time: ***β* = .137, SE = .020** ^***^
**Author/s**	**Year**	**Sample**			**Test of IPV**	**Test of depression**	**Type of IPV**	**Depression finding/s**
		**Location (Country)**	**Total females (N)**	**Source**				
Oh et al.	2019	Korea	3732	Nationally representative sample from the *Korea Welfare Panel Study*	KWPS	CESD‐11	Any	A new exposure to IPV had a positive association to the onset of depressive symptoms in females without depression at baseline: ***β* = 1.594, SE = .183** ^***^
Ouellet‐Morin et al.	2015	UK	978	Participants from the *Environmental Risk Longitudinal Twin Study*	CTS	DIS	Any	Females without a history of depression who experienced IPV at 33years had increased odds of new onset depression at: 38 years: ***aOR* = 1.72 (95% CI: 1.07, 2.77)** ^*^ 40 years: ***aOR* = 1.61 (95% CI: 1.05, 2.48)** ^*^
Simmons et al.	2018	USA	466	Nationally representative sample from the *National Youth Survey Family Study*	CTS	DIS	Any	Exposure to IPV at baseline was not predictive of depression diagnosis at follow up: *β* = −0.05, SE = .40, *p* = 0.91
Watkins et al.	2014	USA	375	Data from a study on *Emotion Dysregulation and Sexual Revictimization Among Young Adult Women* in the community.	CTS	DASS	Physical Psychological	Symptoms of depression Never experienced physical IPV vs ever experienced: *β* = .194, SE = 0.087 ^*^ Never experienced psychological IPV vs ever experienced: *β* = .020, SE = 0.101

Note: ^*^p < .05, ^**^ *p* < .01, ^***^ *p* < .001,.

Effect estimates in bold were included in the meta‐analysis. Abbreviations: AAS, abuse assessment screen (Soeken et al. 1998); CAS, composite abuse scale (Hegarty, Sheehan, and Schonfeld 1999); CESD‐10, centre for epidemiologic studies depression scale ‐ 10 item (Irwin, Artin, and Oxman 1999); CESD‐11, Centre for Epidemiologic Studies Depression Scale ‐ 11 item (Park and Kim 2020); CIDI, composite international diagnostic interview (Wittchen 1994); CTS, conflict tactic scale (Straus et al. 1996); DASS, depression anxiety and stress scale (Lovibond and Lovibond 1995); DIS, diagnostic interview schedule; GDS, Goldberg anxiety and depression scale (Goldberg et al. 1988); KWPS, Korea Welfare Panel Study (Park et al. 2017); MFQ, moods and feelings questionnaire (Angold et al. 1995); PHQ‐9, patient health questionnaire (Kroenke, Spitzer, and Williams 2001); PROVIDE, PROVIDE questionnaire (Yakubovich et al. 2019); WHOMCS, World Health Organization Multi‐Country Study on Women's Health and Domestic Violence (García‐Moreno et al. 2005).

## 3 Results

### 3.1 Description of Studies

The search protocol produced 269 studies from the *Medline* database, 562 studies from the *PsychInfo* database, and 362 studies from the *Ebscohost* database, producing a total of 1193 studies. The authors’ names, study titles, and abstracts were entered into a spreadsheet, and, with the removal of duplicates, the total number of studies was reduced to 748. Study titles and abstracts were evaluated for alignment with the research topic by the first author, and a further 654 studies were excluded. The factors assessed for the removal of studies at that stage included the absence of measurement of one or both IPV and depression, the study was not written in English or depressive symptoms were only measured at a single timepoint. Following this process for elimination of non‐relevant studies, exclusion, non‐exclusion, and inclusion criteria were applied to the remaining 94 studies, and, as a result, 11 longitudinal studies remained for analysis. The full text version of each study was retrieved, and these were evaluated by both authors blindly. It was determined that all 11 studies met the criteria for inclusion in the review, and the descriptive characteristics of these studies are summarized in Table 1.

### 3.2 IPV Measurement

There was variation in the number of times IPV was assessed in the 12 studies included in this review. One study measured IPV at one timepoint, four studies at two timepoints, three at three timepoints, two at four timepoints, and one at six timepoints. All of the reviewed studies assessed IPV at the commencement of the study (referred to here as baseline). The most frequently used method to measure IPV was the conflict tactics scale (CTS; Straus et al. 1996). The CTS is a series of five self‐report questionnaires that examine the strategies used by an individual to manage conflict in their intimate relationship (Chapman and Gillespie 2019). It has been shown to have satisfactory reliability and validity and is well established as a dependable instrument for the measurement of IPV in research contexts (Chapman and Gillespie 2019). The CTS evaluates all forms of IPV, including physical, sexual, and psychological violence (Chapman and Gillespie 2019). The full version of the scale was administered in two of the studies, while a further three studies adopted only some of the questionnaires.

### 3.3 Depression Measurement

Similar to the measurement of IPV, depression was assessed at differing time points across the 12 studies analyzed for this review. It was measured twice in four studies, three times in three studies, four times in two studies, and six times in one study. All but one of the reviewed studies evaluated depression at baseline. In relation to measurement method, seven studies used self‐report methods and four applied clinician interviews to establish presence and severity of depression. The Centre for Epidemiologic Studies Depression (CESD) self‐report questionnaire was the most commonly used, with three studies administering the 10‐item short form (CESD‐10) and two studies utilizing the 11‐item short form established for Korea (CESD‐11). Both short forms of the CESD have been assessed as having high validity and internal consistency (Irwin, Artin, and Oxman 1999; Park and Kim 2020).

### 3.4 Adjustments for Confounders

The reviewed studies adjusted for various socio‐demographic factors. The most common were sex, age, race, education level, and economic status. One study also adjusted for parental socio‐demographic variables, including racial background, maternal age at pregnancy, maternal marital relationship, maternal education, family income, and maternal depression. Four studies controlled for alcohol use and smoking, two for chronic health conditions, and one for body mass index. The most extensive analysis of covariates was performed by Herbert et al. (2022), who corrected for known risk factors of IPV and depression, including socioeconomic status at birth, ethnicity, whether the participant was part of a sexual minority, anxiety, extreme parental monitoring at age 15, anti‐social behavior at age 14, smoking at age 16, cannabis use at age 16, illicit (non‐cannabis) drug use at age 16, hazardous alcohol use at age 18, adverse childhood experiences from birth to age 16, low self‐esteem at age 17, overweight at age 17, sleep problems at age 17, and parents’ education level. Four of the studies adjusted for childhood maltreatment, which specifically included child sexual abuse in one instance.

### 3.5 Exposure to IPV and Subsequent Depression in Women

**FIGURE 2:**
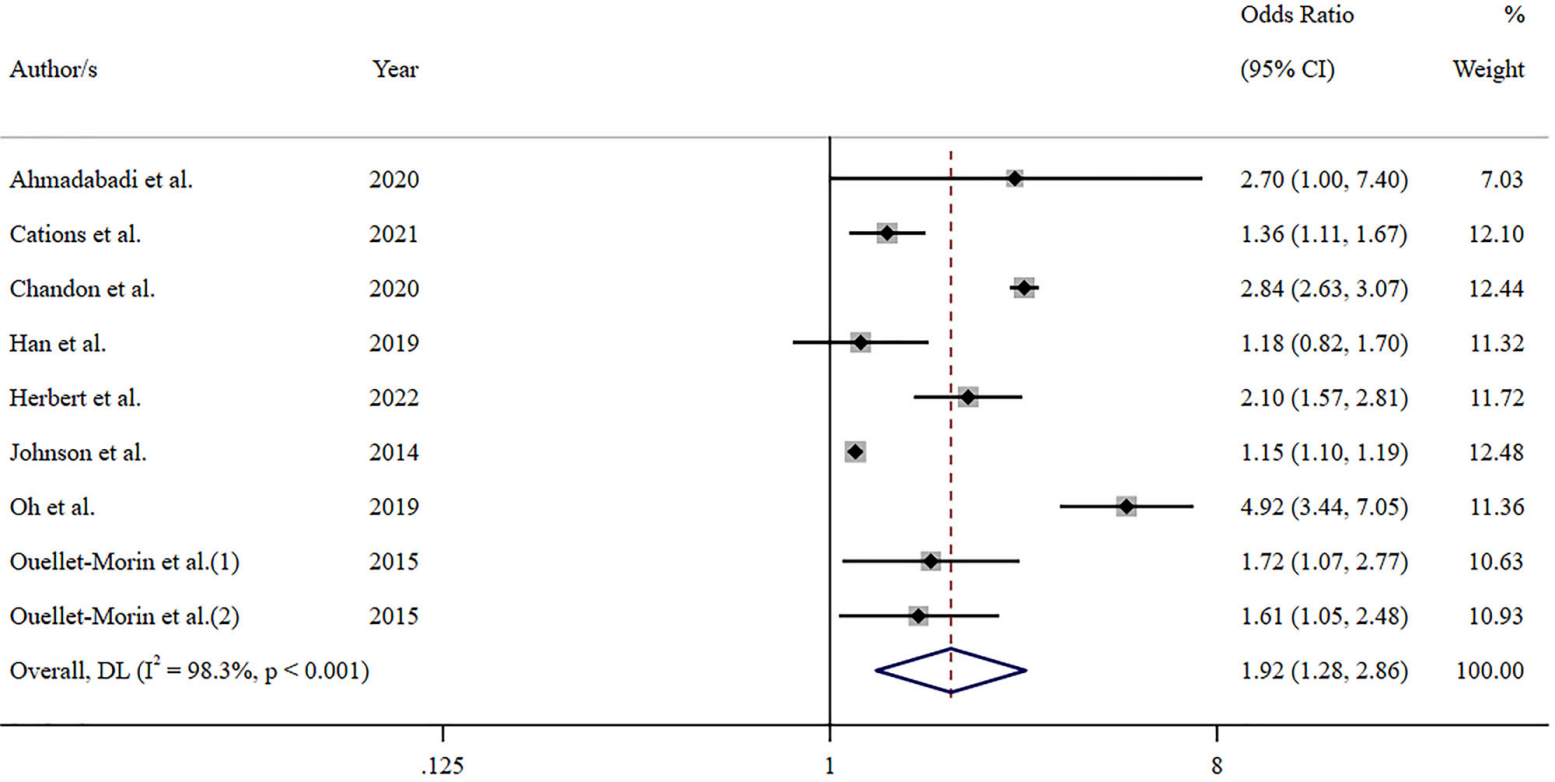
**|** Meta‐analysis of the association between IPV and new onset depression in women.

The reviewed studies reported 22 effect estimates assessing the relationship between exposure to IPV and the subsequent onset of depression in women. Eleven estimates were reported as statistically significant at *p < 0.05*, three at *p* < 0.01, and three at *p* < 0.001. Twelve of the 22 effect estimates examined only one element of IPV, such as physical or sexual violence, while 10 estimates included all three elements: physical, sexual, and psychological violence. Of these 10 effect estimates, nine were from studies that measured depression at baseline and excluded participants with a history of depression from their analysis. These nine estimates were included in the meta‐analysis of the association between IPV and new‐onset depression in women (Figure 2). The pooled odds ratio for these nine estimates was *OR* = 1.92 (95% CI: 1.28, 2.86), which was highly heterogeneous (*I^2^* = 98.3%, *p* < 0.001), although all studies showed a positive direction of effect. The sensitivity analysis demonstrated that the inclusion of all effect estimates in the meta‐analysis was congruent with the results of the main analysis (*OR* = 1.61, [95% CI: 1.25, 2.09], *I^2^* = 96.8%, *p* < 0.001). Findings of the sensitivity analysis are summarized in Supporting Information .

At the level of individual study outcomes, one of the strongest positive associations between IPV victimization and subsequent depression was reported by Chandan et al. (2020) in their study of 92,735 women. That study determined that the risk of developing depression in women exposed to IPV was over 3 times higher than for those in the unexposed group (*IRR* = 3.40 [95% CI: 3.16, 3.67]). Similar findings were reported by Ahmadabadi et al. (2020) in their investigation of 891 Australian women, which assessed the temporal association between exposure to IPV at age 21 years and the subsequent development of depressive symptoms at 30 years. That study found that exposure to any form of IPV at age 21 years almost tripled the odds of developing a depressive disorder at age 30 years after adjusting for parental racial background, maternal age at pregnancy, maternal marital relationship, maternal education, family income, maternal depression education, marital status, having children, and sexual child abuse (*aOR* = 2.70 [95% CI: 1.0, 7.4]).

In their UK study of 1764 women, Herbert et al. (2022) demonstrated that women who experienced IPV between 18 to 21 years of age had on average 26% higher depressive symptom score at age 23 years (*RoGM* = 1.26 [95% CI: 1.13, 1.40]); these women were twice as likely to meet the threshold for diagnosis of a depressive disorder at age 23 years (*OR* = 2.10 [95% CI: 1.57, 2.81]). Comparatively, Chatterji and Heise (2021) found a moderately strong association between IPV exposure and subsequent depression in their study of 1536 Rwandan women. Those authors determined that the experience of physical or sexual IPV at baseline resulted in an almost 50% increase in the relative risk for the development of depressive symptoms at 24 months after baseline (*aRR* = 1.49 [95% CI: 1.13, 1.96]). They also found that the experience of high‐intensity emotional IPV at 12 months was associated with a higher relative risk of depressive symptoms at 24 months than was the case for physical or sexual violence (*aRR* = 1.82 [95% CI: 1.15, 2.89]).

Studies of the Korean population by Oh et al. (2019) and Han et al. (2019) also reported strong positive association outcomes between IPV victimization and subsequent depression. Oh et al. (2019) performed a secondary analysis of retrospective data from 3732 women collected over a 6‐year period. They determined that females who experienced their partner transition from being non‐violent to violent at any time in the 6‐year period of the study had a statistically significant chance of reporting high depression scale scores (*β* = 1.594, SE = 0.183, *p* ≤ 0.0001). Using the same primary dataset, but investigating 3143 participants from a different time period, Han et al. (2019) established that experiencing any type of IPV victimization increased the odds of developing depression by 18% over the 12‐month period of the study (*aOR* = 1.18 [95% CI: 0.82, 1.70]). The researchers also found that sufferers of physical violence as a subset of IPV had almost twice the odds of recording high depressive scores (*aOR* = 1.96 [95% CI: 1.31, 2.95]).

Consistent with these findings, the study by Ouellet‐Morin et al. (2015) concluded that women who had experienced IPV at 33 years of age were one‐and‐a‐half to two times more likely to report the subsequent onset of new depression at 38 years (*aOR* = 1.72 [95% CI: 1.07, 2.77]) and at 40 years (*aOR* = 1.61 [95% CI: 1.05, 2.48]). Notably, that study also examined childhood maltreatment as a covariate and determined that experience of both IPV and early maltreatment resulted in a nearly three‐and‐a‐half times increase in the odds of developing depression at 38 years (*aOR* = 3.43 [95% CI: 1.79, 6.57]). It also resulted in a nearly four‐and‐a‐half times increase two years later at 40 years (*aOR* = 4.30 [95% CI: 2.43, 7.60]).

Cations et al. (2021) assessed the impact of historical IPV on the well‐being and risk for elder abuse in a cohort of older Australian women (70 to 75 years of age). That study found that women who reported a history of IPV had significantly poorer psychological well‐being overall and were particularly vulnerable to incident depression over the follow‐up period of the study (*aOR* = 1.36 [95% CI: 1.11, 1.67]). Comparatively, in an examination of adolescents and young adults, Johnson et al. (2014) established that experiencing IPV victimization at any time during the 6‐year study increased the measures of depressive symptoms regardless of sex (*β* = 0.065, SE = 0.017, *p* < 0.001). They also demonstrated that being a female victim of IPV had a statistically significant positive association with the subsequent development of depressive symptoms during the six years of the study (*β* = 0.137, SE = 0.020, *p* < 0.001). Similarly, Watkins et al. (2014) demonstrated that depressive symptoms were higher in females who experienced physical IPV at any time during a 12‐month period when compared to women who had no experience of physical IPV (*β* = 0.194, SE = 0.087, *p* < 0.05). However, those researchers found that there was no statistical difference between the depression outcomes of women who experienced psychological IPV and those who did not (*β* = 0.020, SE = 0.101).

Finally, of the 11 reviewed studies, Simmons et al. (2018) was the only investigation to report effect estimates that did not suggest a statistically significant positive association between IPV and the onset of depression. However, this study was also the only investigation that did not measure depression at baseline and exclude individuals with a history of depression from the analysis. In this study, the assessment of IPV was divided into minor and major victimization, with the latter distinguished by violent acts such as kicking, biting, hitting, threatening to use a weapon, or using a knife or firing a gun. A multivariate regression analysis was performed that demonstrated that minor victimization did not have a positive association with depression in women (*β* = −0.05, SE = 0.40, *p* = 0.91), while major victimization on the other hand, had a marginally significant association (*β* = 1.00, SE = 0.54, *p* = 0.06). Subsequently, the authors concluded that the hypothesis that IPA victimization is positively associated with negative emotional outcomes was not supported.

## 4 Discussion

The aim of this systematic review and meta‐analysis was to evaluate longitudinal studies published after 2013 that assessed IPV as a contributor to the development of depression in women. Analysis of the 11 studies that formed the basis for this review revealed that 10 reported a statistically significant positive association between exposure to IPV and the subsequent onset of depression in women. The magnitude of this association varied greatly, with the strongest effect estimates reported in studies with large sample sizes. In the current meta‐analysis, 10 effect estimates from nine studies demonstrated that female survivors of IPV had almost double the odds of developing subsequent depression (*OR* = 1.92 [95% CI: 1.28, 2.86]). These findings support the hypothesis that IPV is a contributor to the development of depression in women and are consistent with the results of previous reviews. For example, in their meta‐analysis of six longitudinal studies, Devries et al. (2013) determined that exposure to IPV almost doubled the odds of a new onset of depressive symptoms in female survivors (*OR* = 1.97 [95% CI 1.56–2.48]).

### 4.1 Limitations of the Included Studies

There were some methodological limitations in the evaluated studies that possibly reduced the robustness of the findings they reported. The World Health Organization defines IPV as including physical, sexual, and psychological violence (WHO 2013). However, a number of the reviewed studies defined IPV as physical violence only, or a combination of two subtypes of IPV, such as physical and/or sexual violence, or physical and verbal abuse. Current inconsistent definitions and measurements of IPV limit the ability to make statistically valid and reliable comparisons from existing data. Furthermore, while a number of studies focused on the effects of physical violence, psychological violence is far more common (Dokkedahl et al. 2022). As such, measures focused on physical and sexual violence alone may underestimate the prevalence of IPV and its negative health consequences. In an attempt to avoid this underestimation, this meta‐analysis contained only effect estimates from studies that defined IPV as including physical, sexual, and psychological violence.

A further limitation of the reviewed studies is the inconsistent control for potential confounders. While all studies controlled for socio‐demographic variables, they varied greatly in their control for established predictors of depression such as childhood maltreatment, chronic illness, substance abuse, socio‐economic status, and trauma (APA 2022). Failure to adjust for these confounders does not permit their exclusion as contributors to depression outcomes. However, it is noted that the effect estimates calculated by the studies reviewed here demonstrated a positive association between IPV victimization and the development of depression regardless of the confounders considered. Furthermore, there was not a substantial difference in the magnitude of the positive association between studies that only adjusted for socio‐demographic variables and those that controlled for established predictors of depression. Therefore, it is argued that the positive association between IPV victimization and subsequent depression is unlikely to be entirely accounted for by confounders.

Ten of the 11 reviewed studies were performed on samples from high‐income, industrialized countries, where evidence suggests there is a lower prevalence of IPV (Garcia‐Moreno et al. 2006; Sardinha et al. 2022). Reliance on findings from these sample groups limits their generalizability to the broader population and may underestimate the global health impact of IPV (Sardinha et al. 2022). The studies also varied greatly in sample size, design, and measurement, particularly in the assessment of depression.

### 4.2 Limitations of This Review

This review undertook an extensive search of the literature, and studies were included through the systematic application of inclusion and exclusion criteria and a quality assessment process. Despite this, there are some limitations. The systematic search was restricted by date range and terminology, which may limit the overall estimation of effect. To fully evaluate the effect of the accumulation of evidence, comparisons must be made between this review and earlier meta‐analyses. The reliance on English language studies reduced the number that could be included in the analysis and may also have contributed to the majority of reviewed studies being from high‐income, industrialized countries. The variety of effect estimates used and the differing scales of measurement employed across the studies limit the further examination of the heterogeneity in the meta‐analysis. Nevertheless, all reviewed studies demonstrated a positive direction of association, which was consistent with 10 of the 11 studies reviewed.

### 4.3 Implications for Research and Practice

The findings of this review suggest a statistically significant positive association between IPV victimization and the subsequent onset of depression in women. It has been argued that IPV is a preventable global health problem, and, as such, this review raises awareness of the need for IPV prevention strategies that may result in a subsequent reduction in the prevalence of women's depression (Coker 2006; Ogunsiji and Clisdell 2017). Assessments of the current prevention strategies suggest mixed effectiveness and low replication of positive outcomes (Dutton 2012; Ogunsiji and Clisdell 2017; Whitaker et al. 2013). It is argued this is the result of curriculum targeted at high school students and young adults, which does not adequately translate to the delivery of programs in the wider community setting (Whitaker et al. 2013). It is also attributed to the absence of a universal approach to prevention that considers all cultures and socio‐economic contexts (Whitaker et al. 2013). Research suggests that community‐based services have concentrated on secondary and tertiary prevention programs that focus on reducing recidivism in IPV perpetrators and future avoidance for IPV victims (Ogunsiji and Clisdell 2017; Whitaker et al. 2013). Given the growing burden of both IPV and depression, there is evidence supporting the exploration of primary proactive prevention approaches for the wider community rather than targeting specific cohorts.

In addition to IPV prevention strategies, there is also an opportunity to investigate the effectiveness of depression intervention strategies for female survivors of IPV. These findings suggest that depression screening should be important part of IPV. Current approaches to IPV victimization include counselling and psychological support (Eckhardt et al. 2013; Neave, Faulkner, and Nicholson 2016). However, the efficacy of these treatments is often determined by the frequency of revictimization rather than the mental health outcomes of the individual (Eckhardt et al. 2013). In a review of treatment effectiveness for IPV survivors, Karakurt et al. (2022) reported that cognitive behavioral therapy (CBT), trauma‐based programs, and support initiatives such as telephone social support and victim empowerment programs were not independently beneficial for the treatment of depression. Combinations of these treatments did show a moderately positive effect, particularly those including support initiatives designed to empower the IPV survivor. Given CBT is among the most common psychotherapies used for the treatment of depression (Chand et al. 2018), these findings suggest that victims of IPV may require alternative approaches to depression intervention than traditional models.

While the evidence of this review suggests IPV victimization contributes to the development of depression in women, further longitudinal studies of large population‐based samples are required that have consistent definitions and measurements of IPV and depression. This research should also include a broader consideration and adjustment for the established precursors of depression, such as childhood maltreatment, traumatic life events, chronic illnesses, and substance abuse. Research would also benefit from exploration of more diverse populations and cultures due to the dominance of studies of high‐income Western populations in the current literature. Large‐scale studies in developing countries would contribute significantly towards understanding the magnitude of the association between IPV and depression.

## 5 Conclusion

Despite the significant burden of IPV and depression on women's health globally, and the clear link between traumatic stress and depression, there is limited understanding of the role IPV plays as a contributor to the development of depression. The primary aim of this systematic review and meta‐analysis was to assess the post‐2013 longitudinal evidence of an association between IPV and depression. It was hypothesized that the traumatic stress of IPV victimization would act as a key contributor to the subsequent onset of depressive symptoms in women. Meta‐analysis results suggest some support for this hypothesis; however, inconsistent adjustment for confounders and variability in the definition and measurement of IPV and depression limit firm conclusions.

## Author Contributions

**Christopher B. Watson**: Writing—original draft; writing—review and editing; conceptualization; methodology; investigation; data curation; formal analysis. **Vicki Bitsika**: Supervision; writing—review and editing; conceptualization.

## Conflicts of Interest

The authors declare no conflicts of interest.

### Peer Review

The peer review history for this article is available at https://publons.com/publon/10.1002/brb3.70236.

## Supporting information

Meta‐analysis of all effect estimates examining the association between IPV and its subtypes and depression in women

## Data Availability

The data analyzed for this study are available on request to the corresponding author.
