# Supplementary figures and images for: Intimate Partner Violence and Subsequent Depression in Women: A Systematic Review and Meta‐Analysis of Longitudinal Studies
Source: Brain Behav. 2025 Jan 19;15(1):e70236. doi: 10.1002/brb3.70236 (PMC11744043; doi:10.1002/brb3.70236)

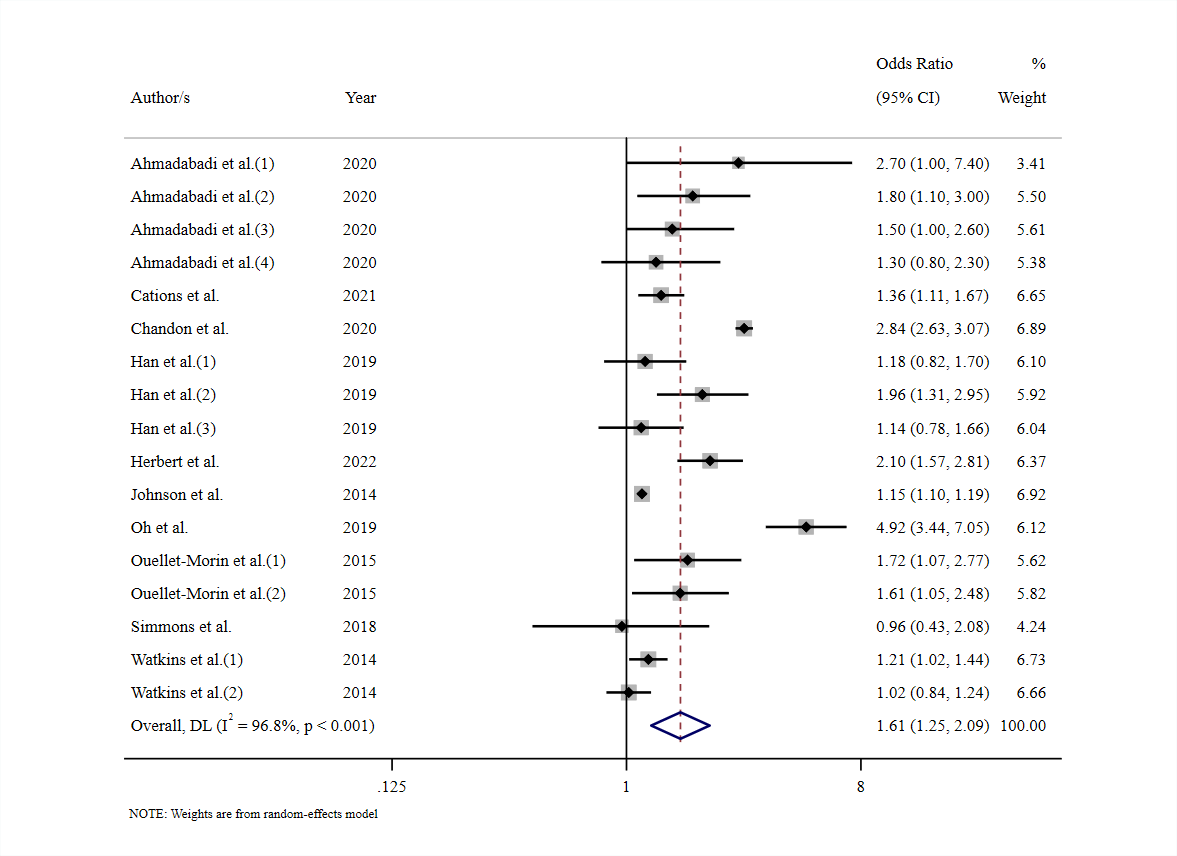

Supplement: Supplementary file 1 — Meta‐analysis of all effect estimates examining the association between IPV and its subtypes and depression in women [file BRB3-15-e70236-s001.png]
